# Social Isolation and Psychosis: Perspectives from People with Psychosis, Family Caregivers and Mental Health Professionals

**DOI:** 10.1007/s10597-022-00941-x

**Published:** 2022-01-25

**Authors:** Penny D. Xanthopoulou, Jennifer Mbanu, Agnes Chevalier, Martin Webber, Domenico Giacco

**Affiliations:** 1 College of Medicine and Health, University of Exeter, Exeter, EX1 2LU UK; 2 Devon Partnership NHS Trust, Dryden Road, Exeter, EX2 5AF UK; 3 Unit for Social and Community Psychiatry, (WHO Collaborating Centre for Mental Health Service Development), Barts and the London School of Medicine, Queen Mary University of London, Newham Centre for Mental Health, London, E13 8SP UK; 4 International Centre for Mental Health Social Research, Department of Social Policy and Social Work, University of York, York, YO10 5DD UK; 5 Division of Health Sciences, Warwick Medical School, University of Warwick, Coventry, CV4 7AL UK

**Keywords:** Psychosis, Isolation, Community, Stigma, Hospitalisation

## Abstract

This paper explores the subjective experiences of mental health practitioners, people with psychosis and carers, on social isolation and community integration of people with psychosis. Focus groups and one-to-one interviews with 80 adult participants across three sites in the UK were conducted. Audio recordings were transcribed and analysed using thematic analysis. Participants commented on various aspects that may cause social isolation or enable community integration, including institutional factors (lack of resources, hospitalisation impact), illness symptoms (e.g., paranoia; over-pathologising vs individual choice), stigma (particularly the psychosis label), and the importance of communities that foster agency and embrace change. Hospitalisation maybe be a cause for isolation and psychiatric wards should consider allowing for socialisation as a therapeutic tool. Initiatives should consider the social fabric of our communities, socioeconomic inequalities and stigmatisation. Building communities that are accepting, kind and flexible can create opportunities that could lead to independence from mental health services.

## Introduction

It is well-known that community-based factors are associated with mental illness (Allen et al., 2014), e.g., having neighbourhood relationships, participating in local activities and schooling (Cheung et al., 2017). In addition, social and self-stigma towards mental illness and the experience of stigmatising attitudes in the community, is a major barrier to social integration (Gonzales et al., 2018; Muñoz et al., 2011). Social integration and feeling a valued member of ones’ community are beneficial both for the individual and communities as a whole (Haldane et al., 2019). Unemployment and austerity also play a role in increasing uncertainty, loneliness and isolation (Moreno et al., 2020). This is particularly relevant to people diagnosed with psychosis, as social isolation and lack of social support play a role in the development of psychosis and the persistence of symptoms (Broome et al., 2005; Giacco et al., 2012; Lee & Seo, 2020). Being socially integrated in one’s community (Galderisi et al., 2015) can generate a stable support network, often cited as crucial to recovery (Wood & Alsawy, 2018). Socially isolated individuals report lower levels of life satisfaction (Bornheimer et al., 2020) and amongst the psychosis population, higher levels of relapse and hospitalisation are also observed (Porcelli et al., 2016; Tee et al., 2020).

Currently, care and support for people with psychosis mainly includes pharmacological interventions, practical support (e.g., help with shopping, medication management and household maintenance) and sometimes talking therapies. Despite these provisions, many remain socially isolated (Giacco et al., 2012). Previous research provides evidence that supporting people with psychosis to engage in social activities in their community through initiatives such as befriending, peer support or through employment and education, could help them increase their social networks (Drake & Whitley, 2014). Most interventions though do not focus sufficiently on the community aspect (Castillo et al., 2019) and relevant initiatives are not part of routine care. Recently however, the National Health Service (NHS) in England (2019) commissioned the Community Mental Health Framework, an integrated model of care that aims to help people living with mental illness to be active participants in their communities, as they see fit, emphasising the importance of social support from family and local communities (Simpson, 2019). In line with studies who distinguish between being locally/community integrated vs having a limited, e.g., family-only social cluster (Harasemiw et al., 2018), we explore this notion of community integration in people with psychosis. Despite the evidence of the impact of social and community integration on people’s mental health, research has primarily focused on individual factors (Cheung et al., 2017).

The recent pandemic and subsequent socioeconomic crises are expected to have a significant effect on people’s mental health (Gunnell et al., 2020; Moreno et al., 2020). Within this context of increased mental illness in the population, lack of resources, and recent efforts to embed mental healthcare in the community, the impact of social isolation and interventions that promote community integration are vital. In this paper, we explored subjective accounts of people with psychosis, their family caregivers and mental health practitioners involved in care delivery in NHS mental health services, on social isolation and barriers/enablers to having social contacts and feeling part of a community.

## Methods

Data were collected between September 2017 and March 2018 as part of large research programme that aims to develop and evaluate an intervention to help people with psychosis to overcome social isolation. Three sites across the UK represented rural and urban areas: East London NHS Foundation Trust, Devon Partnership NHS Trust, and Tees, Esk and Wear Valleys NHS Foundation Trust. Ethical approval was granted by the East of England Cambridgeshire and Hertfordshire Research Ethics Committee (17/EE/0276).

### Participants and Recruitment

**Fig. 1:**
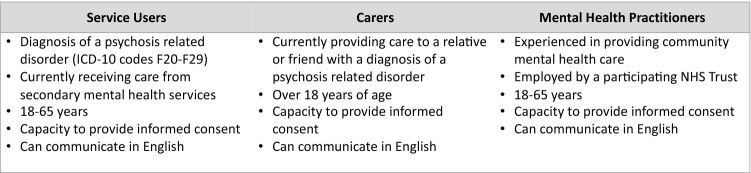
Participant inclusion criteria

Participants were people with psychosis (PwP), family caregivers and mental health practitioners (MHPs), and were identified through secondary care/community mental health services. Carers also self-referred from posters displayed in various outpatient community locations and in participating Trust premises. The study was also promoted at local service user and carer groups. MHPs known to the research team were approached via email or face to face. Informed consent was sought from eligible participants (Fig. 1 inclusion criteria), and included permission for researchers to access their medical records to retrieve socio-demographic information and clinical characteristics. Capacity was assessed at two time points during the study: by MHPs when obtaining assent from interested people with mental illness to be approached by researchers, and by researchers when they were obtaining informed consent.

Focus groups were conducted at each of the three participating Trusts, with each participant group: people with psychosis, carers and MHPs. One to one interviews were conducted with participants who preferred a non-group setting. The focus groups, were facilitated by two researchers and took place across various sites of the participating Trusts. One to one interviews were facilitated by one researcher and took place at either an NHS premise or the participants’ home.

### Analysis

The analysis presented in this paper is part of an interview/focus group study that aimed to understand participants’ views and opinions regarding a psychological intervention to expand the social networks of people living in the community with psychosis. A semi-structured interview guide aimed to elicit participants’ views on the potential benefits of the psychosocial intervention aimed to increase social contacts, possible barriers to engagement and ways the intervention could be improved, e.g. “What might prevent someone from engaging/taking part with this intervention?”. Conversations naturally gravitated towards participants’ experiences of social isolation and community participation. Participant accounts which focused on discussion of isolation and community participation only, were included in the analysis. Accounts relating to specific processes of the intervention (e.g., frequency of sessions, accessibility of the intervention) are published elsewhere (Tee et al., 2020). The focus groups and interviews were audio recorded, transcribed, line-by-line coded using the software program NVivo 12 and analysed using inductive thematic analysis (Terry et al., 2017). One author (PX) coded the data and the initial coding scheme was discussed in analytic meetings with another author (JM). All authors commented on an initial coding frame, which led to the development of the final themes/sub-themes.

## Results

Eighty people participated in 12 groups (6–10 participants) and 9 individual interviews (Table 1), which lasted up to 90 min. The data were thematically organised into 4 main themes (Table 2).

**Table 1:** Participant characteristics

	%
PwP (*N* = 32)	
Mean age (s.d.)	41 (9.8)
Female	44
Carers (*N* = 26)	
Mean age (s.d.)	63 (8.3)
Female	77
Relationship to SU: parent	73
Relationship to SU: spouse/partner	15
Relationship to SU: son/daughter	4
Relationship to SU: sibling	4
Relationship to SU: friend	4
MHP (*N* = 22)	
Mean age (s.d.)	44 (10.0)
Female	59
Years working in mental health, mean (s.d.)	15 (9.5)

**Table 2:** Themes and sub-themes

Themes	Sub-themes
Service pressures and psychiatric hospitalisation as barriers to community integration	▪ Continuing loss of resources in mental health and community services ▪ Long psychiatric hospitalisation as a cause social isolation
Symptoms’ impact on community integration: choice vs pathologising	▪ Losing social network due to symptoms’ attributes and behaviours ▪ Loneliness as symptom vs choice and a decreased sense of community
Isolation result of social and self-stigma	▪ Experience and fear of stigma: embarrassment and diagnostic label ▪ Feeling safe socialising with other people with mental illness
Communities promoting agency and independence from services	▪ Meaningful involvement vs over-prescriptive interventions ▪ Agency: a step between services and independence

## Service Pressures and Psychiatric Hospitalisation as Barriers to Community Integration

### Continuing Loss of Resources

Participants emphasised the loss of resources in mental health services and poor access to limited basic service. They stated that mental health is not a priority for funding and they could see services being steadily dismantled. This impact of this was felt by all stakeholders: “these recent cuts of the services…this particular group of patients are … not part of the mainstream services because they are just, they are socially isolated and unwell somewhere” (MHP).

They also witnessed cuts in community services: “Because you’ve got day centres. But then what do they do? They closed the day centres” (carer). The lack of resources for people recently discharged from in-patient mental healthcare often led to further hospitalisation, police intervention and leaving people unsupported at this crucial part of the recovery and integration back to the community: “people who have recently come out of hospital and are in a bit of a limbo” (PwP).

### Long Psychiatric Hospitalisation as a Cause Social Isolation

In addition to not having resources after hospital discharge, SUs and carers talked how long hospitalisation itself can lead to isolation and limit social interaction. People in mental health hospitals often have no interaction with others for months or years: “The worst thing is not having anything to do and not being allowed out or not having access to people on the outside, it makes you very isolated” (PwP). They described the very limited opportunities in in-patient settings to socialise with others: “that’s when my son started smoking heavily, in hospital… the only social interaction that they have is smoking” (carer), and described how this removal from ordinary life and their sociocultural environment was difficult to overcome when discharged: “He’s been institutionalised for 10 years, so he just doesn’t want to socialise with people” (carer).

## Symptoms’ Impact on Community Integration: Choice Vs Pathologising

### Losing Social Network Due to Symptoms’ Attributes and Behaviours

All participants talked about the various ways symptoms of psychosis impact on socialising and the fact that people with psychosis had lost any previous social networks and friends was apparent to MHPs:

you’ve got anxiety issues and all the stuff that goes with mental health and problems and not had social contact for years (MHP).

a lot of our psychosis folks might have lost all their friends (MHP).

Some described how paranoia affects socialisation: “they don’t necessarily trust their peers” (PwP) and many said that many people with psychosis experience difficulties with change which may stop them from participating in social activities in their communities: “people who are like scared to come out and try something new” (PwP).

This was attributed to either being sectioned for a prolonged period of time: “when you end up in the hospital system and all that, you do lose track of friend from before” (PwP), or because others do not want to associate with people with psychosis: “she would really like it if somebody came to visit her at home because there’s not a single person of her so-called friends in 9 months has come to visit her” (carer). In addition, symptoms can be exhausting and many “are under the influence of antipsychotic drugs” (carer).

### Isolation as Symptom Vs Choice and a Decreased Sense of Community

Talking about symptoms, participants suggested an association between isolation and low confidence, and that prescribing socialisation can increase their motivation to participate in their communities:

Carer A: It’s all about building up confidence… They’re not part of a community. They haven’t got any friends

Carer B: you’re dealing with a group of clientele who have got no motivation

Carer A: It’s getting them out of their bubble isn’t it? Out of their comfort zone

This idea of encouraging people with psychosis to socialise in order to increase motivation, was juxtaposed by SUs and carers who suggested that people might choose to be alone: “I don’t go out, I don’t want to meet people. I just feel safe in here and I don’t want to do that… I’ve got my games, I’ve got virtual reality which is good” (PwP).

This was supported by the idea of a broader decreased sense of community**:** “In a sense this is sort of accepting that we have a society in this country where people live on their own. Rather than as they used to live in a community” (carer). It was suggested therefore, that normalising participation anxiety for people who have been isolated could help them feel less different:

it needs normalising because even you and me, we have high anxiety rates don’t we? (MHP)

just look at your own experience, of going into a new social situation. Bloody terrifying (carer).

## Isolation Result of Social and Self-stigma

### Experience and Fear of Stigma: Embarrassment and Diagnostic Label

Participants from all stakeholder groups talked about low socialisation was sometimes due to people’s experience of stigma and the potential of embarrassment due to their diagnosis: “they might think like ‘oh what if I go and try this activity, what if someone finds out that I’ve got mental illness?’” (PwP). This also applied to carers: “my family disowned me, and people are very estranged when you’re looking after somebody with psychosis” (carer). They described how people in the community don’t know how to respond or behave in relation to psychosis: “we’re aware when people are nervous, embarrassed around us…I think some people are frightened of saying the wrong thing” (carer). This often led to people with psychosis hiding their diagnosis: “say that I’ve got depression rather than schizophrenia because it sounds better” (PwP). Participants also talked about criminalisation due to psychosis symptoms/episodes which can cause inability to participate in the community and education:

I was going to be doing music tech… they wouldn’t accept anyone onto the group with a history of violent crime they said, because I was on a section 37, 41… and it’s six years later and I’ve been doing everything I can and they actually refused me… I tried to convince them I’m not a criminal (PwP).

### Feeling Safe Socialising with Other People with Mental Illness

As a consequence to the stigma felt by people with psychosis, many participants indicated why socialising with other people with psychosis was a better option, as they felt safe with other people with mental illness: “and it’s for people with psychosis … you can just go and have a chat and relax and have a pint without looking over your shoulder … I didn’t feel embarrassed or nervous. You could just be yourself” (PwP). Family caregivers also felt that their loved ones were protected when they socialised with service user groups organised by services, or lived with other people with psychosis, and some believed this was their only option for creating new friendships:

Carer C: “he lives in a bungalow with other people like himself… and then these people who’ve been through similar experiences to my son, some worse, and things and this, and I’m like oh my God these are fantastic, hurrah, they’re a peer supporting group, there’s no judgement, you can be there, you can be safe… And because you don’t have to edit what you’re saying, so it’s a safe environment. They haven’t got any friends, any support. So again if you could get them together and they could get to know the people that were on the group…

Carer D: Someone who hasn’t got this illness they don’t connect. They only connect with the illness. That’s all they know.

## Communities Promoting Agency and Independence from Services

### Meaningful Involvement Vs Over-Prescriptive Interventions

Participants suggested that integration into the community is the best way to recover and live with mental illness. In contrast to protective attitudes that wanted to keep people with mental illness socialising with other people with mental illness only, others described that meaningful participation in the community means relationships with people outside of mental health services, and not fellow services users of MHPs: “it’s about relationship with real people” (carer). This, some suggested, required genuine opportunities to be involved in the community: “activities that are meaningful to the person… something they genuinely enjoy” (PwP), as opposed to over-prescriptive service-led interventions which can lead to loss of agency: “you just have a list, it’s kind of disempowering isn’t it …Going to walk your dog with a friend can be a social activity and that’s not formally organised, that’s more social … not institutional” (MHP).

This ‘list’ can be generic and not meeting SUs’ needs and wishes and therefore becomes redundant: “she doesn’t want to do it… go and do this, let’s go and do that, then it’s pointless doing the care plan” (carer). These care plans were also described as not person-centred, short-term and inflexible: “The client said, oh yes, yes, I want to do something. Then, OK, but it will start in 8 weeks” (MHP).

### Agency: A Step Between Services and Independence

Participants suggested that in order to enable people with psychosis to integrate in their communities, is to increase public understanding of mental illness: **“**there are differences between how much awareness each person has about mental illness and there’s always sometime disagreement and conflict” (carer). Similarly to service care-plans, participants also suggested that community initiatives need to encourage agency and not pathologise difference, but allow it: “it’s about building trust and it’s about feeling comfortable… and knowing that if things get difficult that everybody’s OK if you just remove yourself” (carer). Building communities that are accepting can give people agency by helping to become independent from mental health services: “it would give that sense of independency away from having, to have say a CPN with them all the time” (PwP), and be a step between reliance in mental health services and independence.

## Discussion

Research in the area of recovery from serious mental ill health has mainly focused on individual factors, which less attention given to the impact of community factors (Cheung et al., 2017). In this study we aimed to add to this literature by exploring stakeholder perspectives of the impact of social isolation and their views on what might help, or be a barrier to being integrated in the community. Participant accounts revealed several factors that may cause social isolation or bring about social integration for people with psychosis. These included institutional and illness-triggered factors, and the impact of stigma and discrimination. They pointed to steps and safeguards such as flexible community-based interventions that would promote agency and normalise participation anxiety, as a step between institutionalisation and independence from social services.

In addition to research that demonstrates how hospitalisation may result in new higher distress, trauma or re-traumatisation, which increases symptoms and can cause PTSD (Berry et al., 2013), ‘participants explained how long hospitalisation contributes to social anxiety and causes social withdrawal’ (Chow & Priebe, 2013). A recent study by Smith et al., (2020) also found long hospitalisations to be strongly associated with a decrease in patients’ social integration. Social integration of people who have been institutionalised (Galderisi, et al., 2015) has been described difficult due to the process of hospitalisation, which is usually long and can exacerbate negative symptoms (Abad et al., 2010), especially for people with a history of abuse including increased distress and fear (Frueh et al., 2005). Participants described the pressures of being institutionalised for long periods of time, which often is involuntary (Bird et al., 2020), and detachment from usual living and the impact of seclusion and restraint (Georgieva et al., 2012).

In line with other research (Giacco et al., 2012; Lim & Gleeson, 2014), participants pointed to a circular relationship between symptoms that may cause people with psychosis to isolate and how becoming isolated can exacerbate the psychosis symptoms. For some participants however, isolation was viewed as a result of low confidence/motivation and suggested that people with psychosis should be encouraged to ‘step out of the comfort zone’. Community-based interventions that are person-centred aim to address this using techniques such as motivational interviewing (Rabkin, 2015; Tse et al., 2013). However, discussions also uncovered debates regarding community and broader societal values, how over-pathologising mental illness may mask individual preferences and over-prescriptive initiatives may remove personal choice.

Despite initiatives and interventions to reduce stigma and discrimination around mental illness (Morgan et al., 2018; National Collaborating Centre for Mental Health, 2019), our findings and recent research (Brouwers, 2020; Gonzales et al., 2018) show that these efforts have been largely ineffective. Social/public and self-stigma due to label, symptoms or behaviours was reported by all groups of participants in this study. As a result, people with mental illness have less opportunities to access jobs and education, e.g. due to criminalisation of mental ill behaviour, and less opportunities to socially integrate and connect with diverse groups of people (Corrigan & Watson, 2002; Drake & Whitley, 2014; Muñoz et al., 2011). Caregivers also suggested that they share not only the burden of the illness but often the stigma that accompanies it. Recovery from mental illness is embedded within a complex process of institutionalisation which can enhance discrimination, and within a socioeconomic context where mental illness is associated with deprivation and low social status/social mobility, e.g. high levels of homelessness and victimisation in people with mental illness (Allen et al., 2014; Brouwers, 2020; Gonzales et al., 2018; Teplin et al., 2005).

Participants emphasised the lack of resources in both mental health and community services as a barrier to social integration e.g. lack of available and affordable community activities, and were pessimistic regarding future availability of resources. One reason for the long-stay in psychiatric in-patient units is perhaps the unavailability of resources and facilities in the community (Chow & Priebe, 2013). Initiatives such as *The Community Mental Health Framework* (NHS England, 2019) may be helpful in addressing this by moving recovery to the community. This framework of community mental health provision aims to enable access to people suffering from mental illness, to community resources such as ‘libraries, leisure and social activities’, as well as access to employment and education. This is an important issue in the recovery of people with psychosis which is supported by the higher rates of psychosis in deprived communities (Kirkbride et al., 2017). However, deeper changes in the way public agencies operate within communities, that emphasise social responsibility, need to happen for health equity to be achieved in our societies (Bromley et al., 2018; Castillo & Harris, 2021).

In line with Walker and Thunus (2020), who found that for people with mental ill health, social inclusion was often limited to the mental health settings, we also found that socialisation outside mental health services was difficult for people living with or recovering from serious mental ill health. Participants said people with psychosis often feel unsafe to socialise with people outside mental health services. In line with research (Yanos, 2007) that describes community participation as ‘natural engagement’ with people in the community, participants distinguished between activities in the community with ‘real’ people, and those within institutional settings with other people with mental illness. This juxtaposition of feeling protected within service-led groups and the need for independence from services and *meaningful* integration, can be partly addressed, according to our participants, by people being knowledgeable, sympathetic and flexible regarding the participation of people with mental illness in their communities. This is reflected in other research, that demonstrated the difficulties in engaging with people in the community who are not ‘mental health professionals or their peers’ and calls for efforts to help re-establishing connections with other social systems, to achieve inclusion within communities (Walker & Thunus, 2020).

Slade et al. (2014) identify several misuses and difficulties of the ‘recovery’ concept, e.g., that some patients are not fit for recovery, compulsory detention and effective treatment as the only way to recovery, and misconceptions about the concept of independence and being ‘normal’. They identify connectedness, hope, identity, meaning and empowerment, as key recovery processes. Our analysis of stakeholders’ subjective views and experiences further supports such arguments for better understanding of the concept of recovery and the role community membership, or ‘citizenship’ (Slade et al., 2014), in recovery.

### Limitations and Further Research

The study may have been affected by selection bias as people who participated may be more aware of and favorable to service-led interventions. Most of the focus groups were held on trust premises and involved staff affiliated with services. Similarly, the voices of people with mental illness and family/ caregivers most socially isolated may not be reflected in this analysis as they were the most difficult to approach. Participants were not asked to comment specifically on social integration and the interview focus was on interventions delivered by health services, therefore perhaps limiting the contributions on this topic. However, and despite this, people commented on many issues and causes of social isolation unprompted. Further research could help understand these issues better as well as identifying protective aspects and solutions that are both individual-based (e.g. symptoms management) as well as social and environmental factors. Also, it is not clear if and how initiatives such as the Community Mental Health Framework help with reducing isolation and stigmatisation of mental illness, and further research could explore such initiatives. In this study we did not compare perceptions of the concept of social support/social isolation between the three groups of stakeholders. It would be beneficial to this field for research to explore this.

### Conclusion

Efforts and interventions that aim to support the social integration of people with psychosis within their communities, would need to address the issues identified by people with lived experience, many of whom are ‘left in limbo’ after hospitalisation. Further multidisciplinary research can help develop culturally sensitive interventions that take the family, community and socioeconomic context in account, and how social inequalities that exist within our communities can hinder such efforts. Hospitalisation impact and treatment experiences need to be improved so that is not an additional trauma to overcome.
